# Immune Checkpoint Inhibitors in Malignant Pleural Mesothelioma: Efficacy, Real-World Outcomes, and the Search for Predictive Biomarkers

**DOI:** 10.3390/curroncol33020093

**Published:** 2026-02-03

**Authors:** Giusi Bondì, Serafina Martella, Dimitrios Stylianakis, Alberto Terminella, Filippo Lococo, Alessia Ciarrocchi, Alfonso Fiorelli, Giacomo Cusumano

**Affiliations:** 1 Department of Thoracic Surgery Unit, Policlinico-San Marco Hospital, University of Catania, 95124 Catania, Italy; giusibondi@gmail.com (G.B.); albertoterminella0@gmail.com (A.T.); 2 Respiratory Medicine Unit, Policlinico “G. Rodolico-San Marco” University Hospital, 95123 Catania, Italy; seramartella@gmail.com; 3 School of Medicine, University Hospital and University of Crete, 70013 Iraklion, Greece; dimitrios.stylianakis@gmail.com; 4 Thoracic Surgery Unit, Fondazione Policlinico Universitario “A. Gemelli” IRCCS, 00168 Rome, Italy; filippo.lococo@policlinicogemelli.it; 5 Department of General Thoracic Surgery, Fondazione Policlinico Universitario “A. Gemelli” IRCCS, Università Cattolica del Sacro Cuore, 00168 Rome, Italy; 6 Laboratory of Translational Research, Azienda Unità Sanitaria Locale-IRCCS di Reggio Emilia, 42123 Reggio Emilia, Italy; alessia.ciarrocchi@ausl.re.it; 7 Thoracic Surgery Unit, University of Campania “Luigi Vanvitelli”, 80131 Naples, Italy; alfonso.fiorelli@unicampania.it; 8 Department of General Surgery and Medical Surgical Specialties (CHIRMED), University of Catania, 95124 Catania, Italy

**Keywords:** malignant pleural mesothelioma, immune checkpoint inhibitors, biomarkers, tumor microenvironment, genomic alterations, immunotherapy outcomes

## Abstract

Immunotherapy has significantly reshaped the management of malignant pleural mesothelioma (MPM), offering new therapeutic opportunities after decades in which platinum–pemetrexed chemotherapy represented the only systemic option. However, clinical benefit remains markedly heterogeneous, with outcomes strongly influenced by histologic subtype, patient characteristics, and real-world treatment conditions. Evidence from monotherapy trials has been inconsistent, whereas combination approaches—particularly nivolumab plus ipilimumab—have demonstrated improved survival compared with chemotherapy, mainly in non-epithelioid tumors. Nevertheless, real-world data consistently show lower efficacy and higher toxicity than registrational studies, especially among elderly and unselected populations. Recent translational work has highlighted the relevance of the tumor microenvironment and recurrent genomic alterations such as BAP1, NF2, and CDKN2A in shaping immune activity and potentially modulating response to immune checkpoint inhibitors. Transcriptomic signatures and circulating biomarkers—including soluble mesothelin-related peptide—have shown prognostic associations but no validated predictive value. Overall, current evidence suggests that sensitivity to immunotherapy in MPM arises from a complex interplay of genomic, immunologic, and clinical factors, and that no biomarker is yet suitable for guiding treatment decisions. Prospective studies integrating molecular and immune profiling will be essential to refine patient selection and advance toward a more rationally personalized use of immunotherapy

## Simple Summary

Malignant pleural mesothelioma is a rare and aggressive cancer, often linked to asbestos exposure, for which treatment options have long been limited. Immunotherapy has introduced new opportunities, but its benefits vary widely among patients and are often less pronounced in routine clinical practice than in clinical trials. Outcomes appear to depend on several factors, including tumor subtype, patient characteristics, and the biological features of the tumor microenvironment. Genetic alterations such as BAP1, NF2, and CDKN2A may influence how tumors respond to immunotherapy, yet no biomarker is currently reliable enough to guide treatment decisions. A deeper understanding of how genetic changes, immune activity, and clinical features interact will be essential for developing more personalized therapeutic strategies and improving future outcomes.

## 1. Introduction

**Figure 1:**
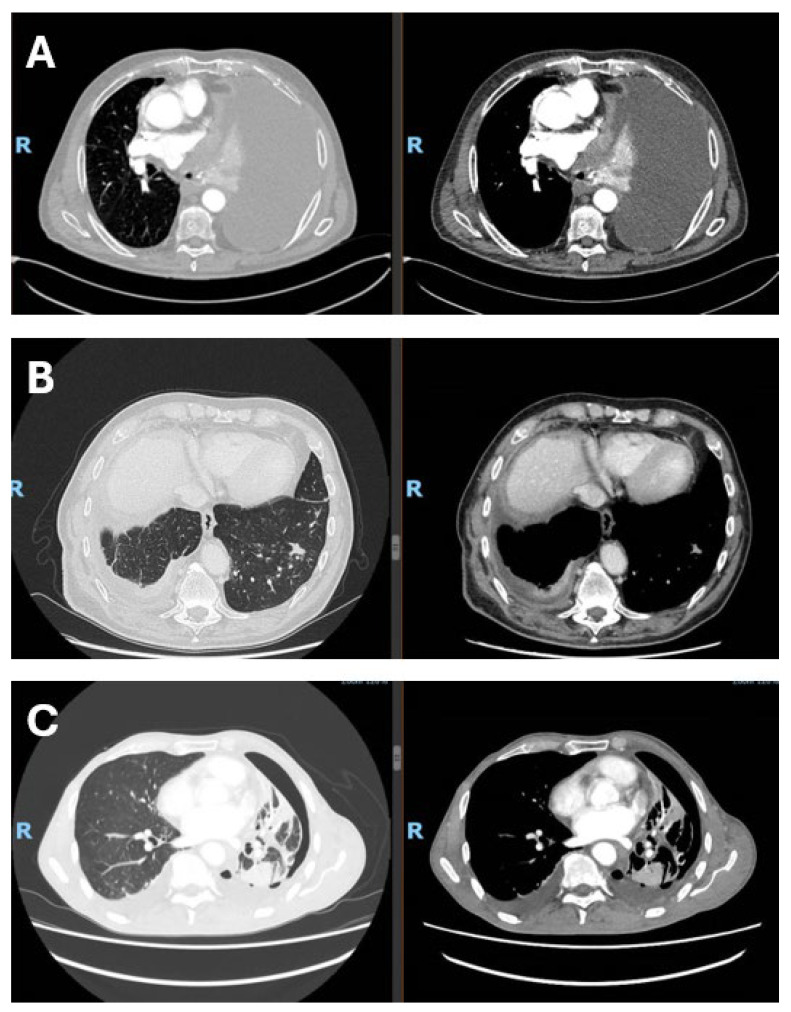
Radiological presentation of malignant pleural mesothelioma subtypes. (**A**) Epithelioid subtype with massive pleural effusion at presentation; (**B**) sarcomtoid subtype with infiltration of the visceral pleura and diaphragm, associated with contralateral metastasis; (**C**) biphasic subtype with visceral pleural involvement and trapped lung. The figure was created by the authors and does not include material subject to copyright.

In this narrative review, we aim to summarize and critically discuss clinical trial data, real-world evidence, and emerging biomarker research on immune checkpoint inhibitors in malignant pleural mesothelioma. Malignant pleural mesothelioma (MPM) is a rare and highly aggressive tumor arising from the mesothelial cells of the pleura and is strongly associated with past asbestos exposure. Owing to its long latency—often exceeding two to three decades—MPM is frequently diagnosed at an advanced stage, when therapeutic options are limited and prognosis remains poor, with median survival generally ranging from 8 to 22 months. Despite asbestos bans in many countries, the global incidence of mesothelioma remains substantial, reflecting historical exposure patterns and the persistent use of asbestos in some regions, with more than 30,000 new cases estimated worldwide each year [1,2]. Asbestos fibers induce chronic inflammation, oxidative stress, and DNA damage within the pleural space, creating a pro-tumorigenic environment that ultimately promotes malignant transformation [3,4]. From a prognostic perspective, MPM remains associated with high mortality, with median overall survival generally below 18 months across population-based and multicenter cohorts, despite variability related to histology, stage, and systemic inflammatory status [5,6]. Real-world studies and national registry data consistently confirm the aggressive natural history of the disease, the heterogeneity of treatment pathways, and persistently limited outcomes despite recent therapeutic advances [7,8]. For many years, therapeutic options were largely confined to surgery, radiotherapy, and platinum–pemetrexed chemotherapy, with no substantial survival gains. Even aggressive multimodal strategies have failed to demonstrate meaningful benefit, as shown by the MARS 2 trial, which reported no improvement—and increased morbidity—with extended pleurectomy/decortication compared with chemotherapy alone [9]. Consequently, MPM remains an area of major unmet clinical need, with primary prevention through elimination of asbestos exposure still representing the most effective strategy. According to the 2021 World Health Organization (WHO) [10] classification of thoracic tumors, MPM includes the epithelioid, sarcomatoid, and biphasic subtypes, as well as preinvasive or low-grade entities such as mesothelioma in situ (MIS) and well-differentiated papillary mesothelial tumor (WDPMT). MIS is defined as a mesothelial proliferation confined to the pleural surface, morphologically similar to reactive processes but characterized by molecular alterations associated with neoplastic transformation. WDPMT is a rare lesion with low malignant potential, characterized by papillary proliferations of mesothelial cells without significant cytological atypia or the molecular alterations typical of malignant mesothelioma, and is associated with an indolent clinical course and favorable prognosis, although rare local recurrences may occur. Among invasive forms, epithelioid mesothelioma is the most frequent subtype and is associated with a relatively more favorable prognosis compared with sarcomatoid and biphasic variants. In this context, the WHO 2021 classification introduced a two-tier grading system (low/high grade) for epithelioid mesothelioma, based on nuclear atypia, mitotic activity, and necrosis, with relevant prognostic implications and the aim of improving interobserver reproducibility and standardizing routine histopathological reporting. Sarcomatoid mesothelioma is associated with the poorest prognosis, while biphasic mesothelioma shows an intermediate clinical behavior, strongly influenced by the proportion of the sarcomatoid component, the increase of which represents a negative prognostic factor [10]. Representative CT images from patients with malignant pleural mesothelioma are shown in Figure 1.

From a biological standpoint, MPM is characterized by a distinctive tumor microenvironment (TME) that plays a central role in disease progression and therapeutic resistance. Although mesothelioma lesions often contain abundant immune infiltrates, antitumor immunity is largely ineffective. T cells display an exhausted phenotype with high expression of inhibitory checkpoints, while immunosuppressive populations such as M2-polarized tumor-associated macrophages, myeloid-derived suppressor cells, and regulatory T cells dominate the TME, collectively fostering immune tolerance [11,12,13]. This immunosuppressive context is further reinforced by epigenetic mechanisms, including EZH2-mediated repression of antigen-presentation and interferon-related pathways, and by the generally low tumor mutational burden of MPM, which limits neoantigen generation and may reduce intrinsic immunogenicity [14,15,16,17]. In parallel, the genomic landscape of mesothelioma is dominated not by oncogene activation but by recurrent loss-of-function alterations in tumor suppressor genes, most notably BAP1, CDKN2A, and NF2 [18,19,20]. These alterations disrupt key cellular processes such as DNA repair, chromatin remodeling, cell-cycle regulation, and Hippo signaling, contributing to genomic instability and shaping both tumor behavior and immune interactions [21,22,23,24,25,26,27,28,29,30,31,32,33]. Increasing evidence suggests that these molecular features may influence immune activity within the TME and potentially modulate sensitivity to immune checkpoint inhibitors (ICIs), although their clinical relevance remains incompletely defined.

Together, the convergence of an immunosuppressive microenvironment and a tumor-suppressor-driven genomic architecture provides a strong biological rationale for the use of ICIs in MPM, while simultaneously explaining the marked heterogeneity in clinical outcomes observed across trials and real-world practice. This complexity highlights a central and still unresolved clinical issue, namely the need to define the clinical contexts, patient subsets, and biomarker frameworks that may allow a more rational and effective use of immune checkpoint inhibitors in malignant pleural mesothelioma.

## 2. Methods

This work is a narrative review aimed at providing a qualitative and critical synthesis of the available clinical, real-world, and translational evidence on immune checkpoint inhibitors in malignant pleural mesothelioma. A comprehensive search of MEDLINE/PubMed, EMBASE, Scopus and the Cochrane Library was conducted for studies on malignant pleural mesothelioma and immune checkpoint inhibitors published up to 20th November 2025. Search strings combined terms for disease (“malignant pleural mesothelioma”, “pleural mesothelioma”), ICIs and drugs (“immune checkpoint inhibitor”, “PD-1”, “PD-L1”, “CTLA-4”, “nivolumab”, “ipilimumab”, “pembrolizumab”, “chemo-immunotherapy”), treatment setting (“first-line”, “second-line”), and biomarkers/TME features (“BAP1”, “NF2”, “CDKN2A”, “PD-L1 expression”, “tumour mutational burden”, “soluble mesothelin”, “tumour-immune microenvironment”, “CD8 T cells”). Searches were limited to human, English-language publications. Reference lists of key trials and reviews, and recent ASCO, ESMO, IASLC and AACR abstracts were also screened.

We included phase I–III ICI trials (monotherapy, dual ICI, chemo–ICI), prospective and retrospective real-world cohorts of ICI-treated MPM, and translational or biomarker studies with clinical outcome data, plus selected preclinical work directly addressing ICI mechanisms. Very small series without meaningful outcome reporting and studies focused solely on non-ICI systemic therapy were not systematically summarised. Study selection and extraction were performed iteratively by the authors, focusing on regimen, line of therapy, population and histology, survival outcomes, key toxicities and biomarker analyses. Because of marked heterogeneity in design, endpoints and reporting, we did not perform formal risk-of-bias assessment or meta- analysis and instead synthesised findings qualitatively, separating randomized trials, real-world evidence and biomarker/preclinical data. Generative artificial intelligence tools were used solely to support language editing and stylistic improvements. No AI-generated content contributed to the scientific analysis, data interpretation, study design, or conceptualization of the manuscript.

## 3. Clinical Evidence and Its Limitations: Trials vs. Real-World Practice

**Figure 2:**
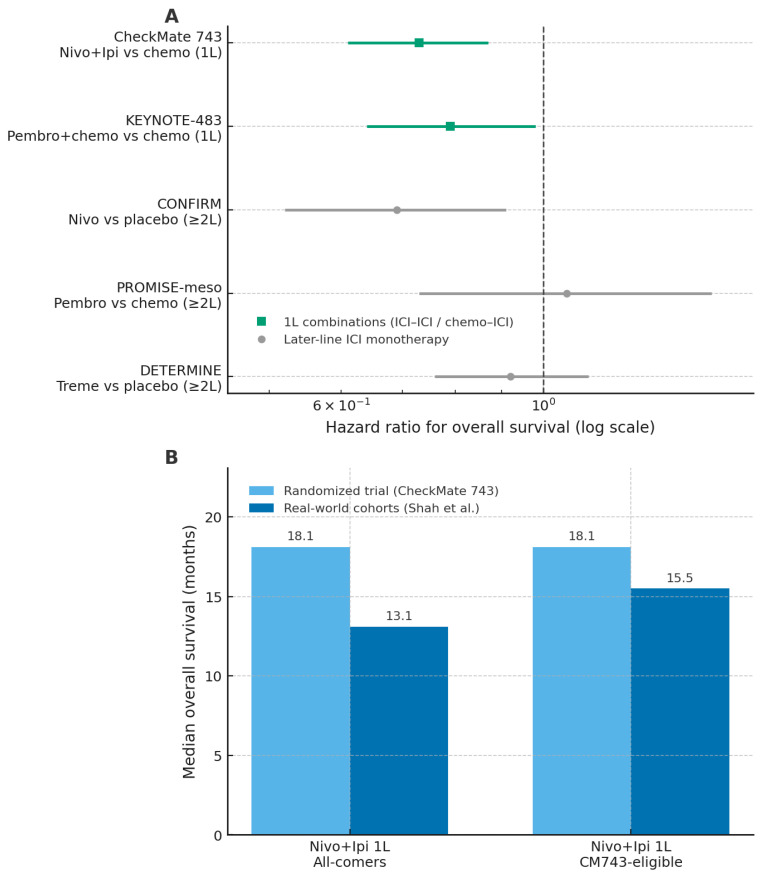
Randomized trials and real-world cohorts of immune checkpoint inhibitors in malignant pleural mesothelioma. Panel (**A**) shows hazard ratios for overall survival from major randomized trials of immune checkpoint inhibitor (ICI) regimens versus their respective control arms, with first-line combinations and later-line monotherapy distinguished by symbol and colour. Panel (**B**) presents median overall survival for first-line nivolumab–ipilimumab in the CheckMate 743 trial and in real-world cohorts, stratified by all-comer and CM743-eligible populations. Abbreviations: ICI, immune checkpoint inhibitor; OS, overall survival; 1L, first line; CM743, CheckMate 743; nivo, nivolumab; ipi, ipilimumab; pembro, pembrolizumab; treme, tremelimumab. The figure was created by the authors and does not include material subject to copyright.

In recent years, immunotherapy has significantly reshaped the therapeutic landscape of malignant pleural mesothelioma (MPM), after decades in which platinum–pemetrexed chemotherapy represented the only available systemic treatment [34]. Nevertheless, evidence on immune checkpoint inhibitors (ICIs) in MPM spans first-line combination regimens, later-line monotherapy, and real-world clinical practice, with heterogeneous clinical outcomes. An overview of randomized trials and real-world outcomes across these treatment settings is provided in Figure 2.

### 3.1. First-Line ICI-Based Regimens

The most relevant advance in first-line treatment emerged from combination strategies. In the phase III CheckMate 743 trial, nivolumab plus ipilimumab demonstrated superior overall survival compared with standard platinum–pemetrexed chemotherapy (18.1 vs. 14.1 months), with a particularly pronounced benefit in non-epithelioid subtypes [35]. In contrast, the survival advantage was marginal or absent in epithelioid tumors, highlighting histology as a critical determinant of benefit. Strategies combining ICIs with chemotherapy have also shown encouraging activity. Phase II studies such as DREAM and PrE0505 reported response rates approaching 50% and median overall survival exceeding historical controls [36,37]. More recently, the phase III KEYNOTE-483 trial confirmed a modest but statistically significant improvement in overall survival with pembrolizumab plus chemotherapy compared with chemotherapy alone (17.3 vs. 16.1 months; HR 0.79) [38]. Despite these positive results, the absence of direct comparisons between dual-ICI and chemo–ICI approaches limits the ability to define the optimal first-line strategy. Moreover, a Journal of Thoracic Oncology editorial highlighted methodological limitations in registrational trials, including informative censoring, differential dropout, and discrepancies between overall and progression-free survival, which collectively weaken the robustness of the conclusions [39].

### 3.2. Later-Line ICI Monotherapy

Earlier trials evaluating ICIs as monotherapy in relapsed or refractory MPM yielded heterogeneous and generally modest results. The DETERMINE trial failed to demonstrate a survival advantage for tremelimumab compared with placebo [40], while the PROMISE-meso study reported a higher response rate with pembrolizumab than with chemotherapy (22% vs. 6%) without an improvement in overall survival [41]. A more consistent signal of activity was observed in the CONFIRM trial, in which nivolumab improved overall survival compared with placebo (10.2 vs. 6.9 months), supporting a selective role for monotherapy in later-line settings [42]. Overall, these data suggest that ICI monotherapy may benefit a subset of patients but does not represent a broadly effective strategy in unselected populations.

### 3.3. Real-World Evidence

Real-world studies consistently report lower effectiveness and higher toxicity of ICIs compared with registrational trials, underscoring the gap between efficacy and effectiveness. In the international MesoNet registry, first-line nivolumab–ipilimumab was associated with a median overall survival of 13.1 months, substantially lower than the 18.1 months reported in CheckMate 743, with minimal benefit observed in epithelioid tumors [43]. Real-world progression-free survival was also inferior (4.2 months), and elderly patients (>75 years), who are underrepresented in clinical trials, experienced particularly limited benefit, with a median overall survival of 9.4 months. Tolerability proved more challenging in routine practice, with immune-related adverse events reported in 37% of patients and grade 3–4 toxicities occurring in 58%, frequently leading to treatment discontinuation [43]. Consistent findings were reported in the multicenter Turkish Oncology Group study, which did not demonstrate significant differences between immunotherapy and chemotherapy in terms of response or survival, except for a modest advantage in later-line settings [44]. Together, these real-world data highlight the limited generalizability of trial results to unselected patient populations and reinforce the need for careful patient selection and more personalized therapeutic strategies. Overall, although immunotherapy represents an important therapeutic advancement in MPM, its clinical benefit appears selective and context-dependent. The variability in outcomes across treatment settings, the reduced tolerability observed outside clinical trials, and the methodological limitations of existing evidence emphasize the need for a cautious and individualized approach to the use of ICIs in routine practice.

Alongside systemic therapies, the role of locoregional approaches and cytoreductive surgery in malignant pleural mesothelioma remains controversial [45,46]. The combination of surgery with hyperthermic intrathoracic chemotherapy (HITHOC) has been associated with acceptable local control and tolerable toxicity in highly selected patients [47,48], but current data do not demonstrate a clear survival benefit [48,49]. Experimental evidence supports the concept that tumor burden reduction and intrathoracic hyperthermia may favorably influence the immune microenvironment and enhance immune checkpoint inhibition, providing a biological proof of principle rather than a clinically validated strategy [48,49]. In this context, mechanical tumor debulking may theoretically facilitate systemic therapies by decreasing overall tumor burden, although this hypothesis remains unproven in clinical practice [49]. Consistent with the results of the MARS 2 and EORTC-1205 randomized trials, critical analyses indicate that cytoreductive surgery should not be adopted routinely and may be considered only in carefully selected cases within multidisciplinary management in high-volume centers [45,46].

## 4. Predictive Biomarkers for Immune Checkpoint Inhibitors

The response to immune checkpoint inhibitors (ICIs) in malignant pleural mesothelioma (MPM) is highly heterogeneous, and no biomarker has yet demonstrated sufficient robustness to guide treatment selection in routine clinical practice. Current candidates span multiple biological levels—including genomic alterations, transcriptomic signatures, protein expression, circulating markers, and immune-cell composition—each supported by variable degrees of mechanistic and clinical evidence.

### 4.1. Genomic Alterations

Recurrent loss-of-function alterations in BAP1, NF2, and CDKN2A represent the most extensively investigated genomic candidates. From a biological perspective, BAP1 and NF2 loss has been associated with a more inflamed and immune-active tumor microenvironment, whereas CDKN2A deletion is linked to immune desertification and impaired lymphocyte recruitment. Clinically, retrospective analyses of ICI-treated cohorts suggest improved outcomes in BAP1- or NF2-deficient tumors and poorer survival in CDKN2A-deleted cases [50,51,52,53]. However, the evidence remains retrospective and exploratory, with no prospective validation or biomarker-stratified trials to date.

### 4.2. Transcriptomic Signature

Transcriptomic approaches have identified gene-expression signatures integrating immune, stromal, and proliferative signals that define prognostically distinct subgroups. A multigene model derived using machine-learning algorithms showed prognostic separation in MPM cohorts [54]; however, these signatures were not developed or validated specifically in ICI-treated populations. As such, current evidence is limited to exploratory and prognostic associations rather than predictive value for immunotherapy benefit.

### 4.3. PD-L1 Expression and Tumor Mutational Burden

PD-L1 expression and tumor mutational burden (TMB), widely used biomarkers in other malignancies, have shown limited clinical utility in malignant pleural mesothelioma. PD-L1 expression reflects interferon-driven signaling and adaptive immune resistance within the tumor microenvironment; however, clinical benefit from immune checkpoint inhibitors has been observed across both low and high PD-L1–expressing tumors, with inconsistent correlations with survival outcomes. Exploratory analyses from ICI-treated cohorts, including CheckMate 743 and PROMISE-meso, as well as first-line chemo-immunotherapy studies such as KEYNOTE-483, have failed to identify PD-L1 expression as a reliable predictor of benefit from immune checkpoint inhibition [35,38,41]. Large multicenter translational analyses conducted in immunotherapy-naïve malignant pleural mesothelioma cohorts have further shown that high tumor-cell PD-L1 expression is an independent negative prognostic factor, supporting its interpretation as a marker of tumor aggressiveness and immune microenvironmental features rather than a predictive biomarker for immunotherapy response [55]. Similarly, tumor mutational burden is generally low in mesothelioma compared with other cancers that are highly responsive to immunotherapy [56] and has not demonstrated a consistent or reproducible association with response or survival under immune checkpoint inhibition, as also discussed in mesothelioma-focused translational analyses of immune resistance mechanisms [57]. For both PD-L1 expression and TMB, the available evidence derives predominantly from retrospective analyses and exploratory correlative studies, and neither biomarker is currently recommended for routine treatment selection in clinical practice. Across the clinical and translational studies discussed, PD-L1 expression has been assessed by immunohistochemistry on tumor cells and reported as the percentage of PD-L1–positive neoplastic cells, corresponding to a tumor proportion score (TPS).

### 4.4. Circulating Biomarker

Among circulating biomarkers, soluble mesothelin-related peptide (SMRP) is the most extensively investigated in malignant pleural mesothelioma and remains the only blood-based biomarker cleared for clinical use in disease monitoring. Baseline SMRP levels correlate with disease burden and extent, and longitudinal changes may reflect disease progression or response to therapy, supporting its role as a monitoring and prognostic marker. However, analyses in immunotherapy-treated cohorts have demonstrated that elevated baseline SMRP is associated with poorer overall survival without correlating with radiologic response or immunotherapy-specific benefit, indicating a prognostic rather than predictive role for immune checkpoint inhibition [58,59]. In addition to SMRP, fibulin-3 has been widely investigated as an alternative circulating protein biomarker in malignant pleural mesothelioma. Recent prospective data evaluating fibulin-3 levels in plasma and pleural effusion have confirmed higher concentrations in patients with mesothelioma compared with non-malignant controls, but with limited sensitivity and substantial inter-study variability, supporting a potential diagnostic or prognostic role rather than clinical applicability for treatment selection [60]. Similarly, osteopontin has been reported to be elevated in patients with malignant pleural mesothelioma and associated with disease burden or prognosis in selected cohorts; however, limited specificity and the absence of validation in immunotherapy-treated populations preclude its use as a predictive biomarker for immune checkpoint inhibition [59,61]. Circulating microRNAs represent an additional area of interest as minimally invasive biomarkers. Several individual plasma microRNAs, including miR-126, miR-103a-3p and miR-625-3p, have been associated with diagnostic or prognostic features in small retrospective series [62]. More recently, mesothelioma-specific studies have proposed circulating microRNA signatures for risk assessment and prognostic stratification, particularly in epithelioid disease, using discovery and validation approaches [63]. However, methodological heterogeneity, limited cohort sizes and lack of assay standardization have hindered reproducibility, and no evidence currently supports a predictive role for circulating microRNAs in selecting patients more likely to benefit from immune checkpoint inhibition [59].

### 4.5. Immune Infiltrate and Epigenetic Regulation

**Figure 3:**
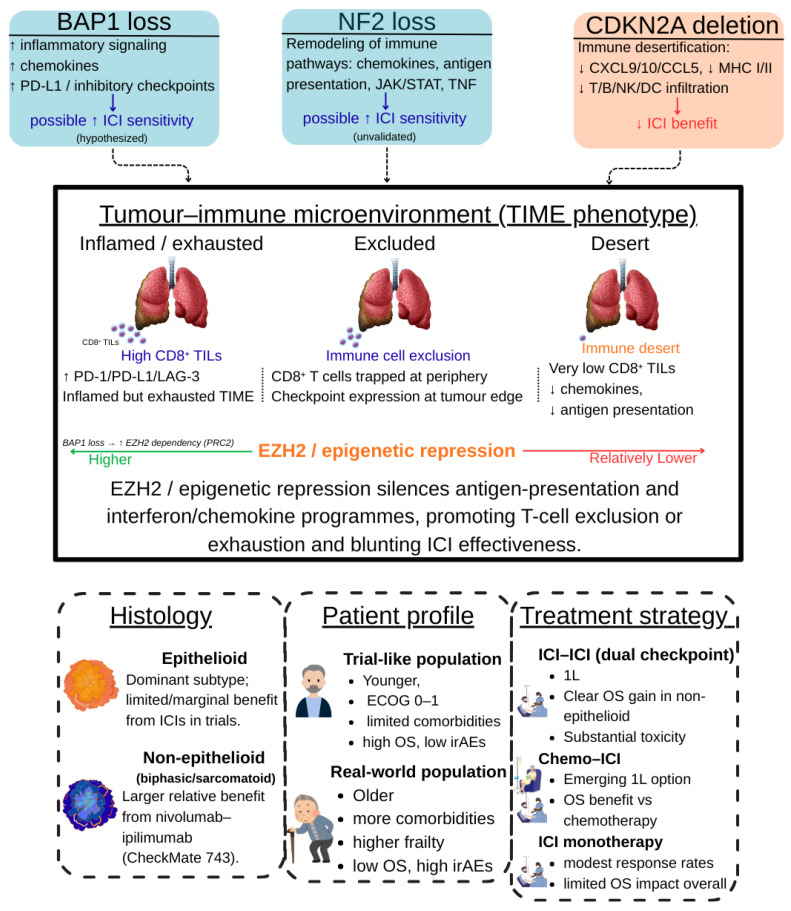
**Integrated determinants of benefit from immune checkpoint inhibitors in malignant pleural mesothelioma**. The schematic summarizes how tumour genotype (BAP1, NF2, CDKN2A alterations), the tumour–immune microenvironment (TIME) (inflamed/exhausted, excluded, desert phenotypes), and clinical context (histology, patient profile, and treatment strategy in trial vs. real-world settings) jointly shape response to ICI. BAP1 and NF2 loss are linked to more immune-active, checkpoint-rich states and are hypothesised to confer higher ICI sensitivity, whereas CDKN2A deletion is associated with immune desertification and lower expected benefit. EZH2-mediated epigenetic repression dampens antigen-presentation and interferon/chemokine programmes, favouring T-cell exclusion or exhaustion and contributing to the efficacy–effectiveness gap observed between clinical trials and routine practice. Abbreviations: MPM, malignant pleural mesothelioma; ICI, immune checkpoint inhibitor; TIME, tumour–immune microenvironment; TILs, tumour-infiltrating lymphocytes; OS, overall survival; irAEs, immune-related adverse events; ECOG PS, Eastern Cooperative Oncology Group performance status; MHC, major histocompatibility complex. The figure was created by the authors and does not include material subject to copyright.

The composition of the tumor immune infiltrate represents another area of interest in malignant pleural mesothelioma. Higher densities of CD8^+^ tumor-infiltrating lymphocytes have been consistently associated with more favorable outcomes and improved responses to immunotherapy across tumor types, including mesothelioma [64]. Conversely, an increased presence of regulatory T cells and myeloid-derived suppressor cells has been linked to an immunosuppressive tumor microenvironment and poorer prognosis, with recent mesothelioma-specific analyses highlighting the prognostic relevance of T-cell ratios within the pleural tumor milieu [65]. More broadly, dysregulation of the immune microenvironment, characterized by suppressive cellular populations and impaired effector function, has been implicated in primary and acquired resistance to immunotherapy in mesothelioma [57]. These observations are largely derived from small and heterogeneous cohorts and remain primarily prognostic rather than predictive. In parallel, epigenetic mechanisms also contribute to immune escape. Preclinical studies have shown that hyperactivity of EZH2 represses antigen-presentation machinery and interferon-related pathways, limiting tumor immunogenicity and promoting resistance to immune checkpoint blockade [66]. Recent integrative reviews further highlight the pleiotropic and context-dependent effects of EZH2 on both tumor cells and the immune microenvironment, underscoring that evidence supporting its role as a predictive biomarker remains predominantly preclinical and insufficient for clinical stratification [67]. Taken together, although multiple candidate biomarkers for ICIs in MPM are supported by biologically plausible mechanisms and emerging retrospective data, none has achieved the level of validation required for routine clinical use. As summarized in Table 1, current evidence is largely preclinical or exploratory, underscoring the need for prospective studies integrating genomic, transcriptomic, immune, and clinical parameters to enable reliable biomarker-driven patient selection (Figure 3).

## 5. Conclusions

The introduction of immune checkpoint inhibitors has significantly reshaped the therapeutic landscape of malignant pleural mesothelioma, with improvements documented in randomized trials—particularly with the nivolumab–ipilimumab combination—and more variable results observed in real-world clinical practice. However, the magnitude of benefit remains heterogeneous, especially among elderly patients, epithelioid subtypes, and unselected populations, indicating that immunotherapy is not a uniformly effective solution.

From a practical standpoint, current evidence supports the use of dual ICI therapy as a first-line option primarily in non-epithelioid disease, where the survival benefit appears most pronounced, whereas its advantage in epithelioid tumors is limited and should be weighed carefully against toxicity. Chemo–ICI combinations offer a modest survival improvement in the first-line setting across histologies but with smaller absolute gains. In later lines, ICI monotherapy may be considered for selected patients with preserved performance status, although expectations regarding benefit should remain cautious. These considerations highlight the need for individualized treatment decisions based on histology, clinical context, and patient fitness rather than a one-size-fits-all approach. In this context, locoregional strategies and cytoreductive approaches have been explored as complementary options in highly selected patients, although current evidence does not support their routine use. Their potential integration with systemic therapies remains investigational and should be confined to multidisciplinary decision-making in experienced centers. From a biological perspective, numerous studies have highlighted the role of specific genomic alterations such as BAP1, NF2, and CDKN2A in shaping the tumor immune microenvironment and potentially influencing response to ICIs. Nevertheless, despite intriguing mechanistic and retrospective clinical associations, none of these factors has achieved sufficient validation to guide patient selection in routine practice. Similarly, circulating biomarkers such as soluble mesothelin-related peptide remain primarily prognostic indicators without demonstrated predictive value for immunotherapy response.

This review has inherent limitations, as it is a narrative review and is based on heterogeneous datasets including randomized clinical trials, retrospective analyses, and real-world cohorts with differing designs, populations, and endpoints. These limitations reflect the current state of the field and underscore the challenges in translating trial efficacy into real-world effectiveness. Future progress will require dedicated efforts in several priority areas: the development of biomarker-integrated clinical trials to prospectively validate genomic and immune predictors of benefit; improved inclusion of elderly and frail patients to better reflect real-world populations; strategies to overcome resistance in biologically unfavorable contexts such as CDKN2A-deleted, immune-desert tumors; and integrative approaches combining molecular, immunologic, and clinical data to enable more precise patient stratification. Only through such coordinated efforts will it be possible to move from heterogeneous immunotherapy outcomes toward a rationally personalized treatment strategy for malignant pleural mesothelioma.

## Figures and Tables

**Table 1:** Candidate biomarkers for immunotherapy in malignant pleural mesothelioma: biological. rationale, evidence for association with ICI outcomes, and current clinical applicability.

Biomarker/Signature	Type	Biological/Immunologic Rationale	Evidence in ICI-Treated MPM	Level of Evidence	Current Clinical Role	Ke Limitations/Open Questions
**BAP1 loss**	Genomic, tumour tissue	Chromatin remodelling; linked to inflamed, exhausted TIME	Retrospective nivolumab–ipilimumab series suggest improved survival	Moderate, exploratory	Candidate predictive; not clinically validated	Small cohorts; heterogeneous assays; no prospective validation
**NF2 alteration**	Genomic, tumour tissue	Alters immune pathways, chemokines, antigen presentation programs	Retrospective data suggest better outcomes with ICIs	Moderate, exploratory	Candidate predictive; needs further confirmation	Few NF2-mutant cases; mixed treatment lines, regimens
**CDKN2A deletion**	Genomic, copy-number loss	Immune desertification; reduced chemokines, MHC, immune infiltrate	Worse outcomes under ICIs in retrospective cohorts	Moderate, negative predictive signal	Adverse prognostic marker; not treatment-exclusion criterion	No stratified trials; assay variability; threshold uncertain
**Transcriptomic risk signatures**	Transcriptomic gene panels	Integrate immune, stromal, proliferation signals; risk groups	Prognostic separation; not designed as ICI predictors	Low–moderate, prognostic only	Research prognostic tools; no role in selection	Derived from mixed therapies; unstandardised platforms, cut-offs
**PD-L1 expression**	IHC, tumour protein	Reflects interferon signalling, adaptive immune resistance	Responses seen across low and high PD-L1	Low, inconsistent predictive value	Descriptive only; not recommended for decision-making	Variable antibodies, scoring, cut-offs; small subgroups
**Tumour mutational burden (TMB)**	Genomic, panel or WES	Generally low; limited neoantigen load in MPM	No clear association with ICI benefit	Low	No established role	Narrow TMB range; few harmonised ICI datasets
**Soluble mesothelin-related peptide (SMRP)**	Circulating serum biomarker	Reflects mesothelin expression and tumour burden	Higher baseline associated with poorer survival on ICIs	Moderate, prognostic only	Prognostic marker; not predictive for ICIs	Single-cohort evidence; assay variability; unclear monitoring value
**Fibulin-3**	Circulating biomarker	Reflects tumor burden	No data supporting association	Low	Diagnostic/prognostic research marker	Limited sensitivity; inter-study heterogeneity
**Osteopontin** **(OPN)**	Circulating biomarker	Reflect inflammatoy signalling	Not evaluated as predictor in ICI-treated MPM	Low	Prognostic research marker	Poor specificity; no immunotherapy validation
**Circulating microRNAs**	Circulating biomarker	Reflect tumour biology and host response; composite risk stratification	Prognostic associations (epithelioid subtype); no evidence of ICI prediction	Low–moderate, exploratory	Research only	Small cohorts; lack of reproducibility; need for prospective validation and assay standardisation
**Immune infiltrates (CD8^+^ TILs, Tregs, MDSCs)**	IHC/multiplex phenotyping	CD8^+^ TILs favourable; suppressive cells worsen immunity	Multiple small series link subsets to survival	Low–moderate, mainly prognostic	Research markers; not standardised clinically	Sampling bias; spatial heterogeneity; non-uniform quantification
**EZH2/epigenetic repression**	Epigenetic/pathway activity	Silences antigen-presentation, interferon, chemokine programmes	Preclinical support for resistance; no stratified clinical data	Low, preclinical	Potential target for combinations, not biomarker	No standard assay; interaction with genotype unresolved

## Data Availability

This study is a review of published literature. No new data were created or analyzed, and therefore data sharing is not applicable.
